# Management of Patients With Hepatitis C Virus, Monoclonal Gammopathy of Undetermined Significance, and Multiple Myeloma

**DOI:** 10.1177/2324709617696854

**Published:** 2017-04-26

**Authors:** Alisse Hannaford, David Del Bello, Siyang Leng, Ajai Chari, Ponni Perumalswami, Douglas Dieterich, Andrea Branch

**Affiliations:** 1 Icahn School of Medicine at Mount Sinai, New York City, NY, USA

**Keywords:** hepatitis C, multiple myeloma, monoclonal gammopathy of undetermined significance, direct-acting antiviral

## Abstract

**Background and Aim:** The vast majority of the 2.7 million individuals in the United States who are currently infected with hepatitis C virus (HCV) were born between 1945 and 1965. The median age of these patients in this particular generation at the time of this writing was 55 years. In the general population, older age is a risk factor for multiple myeloma (MM) and other monogammopathies. As the baby boomer population ages, HCV providers are increasingly likely to encounter HCV-infected patients with a monoclonal gammopathy. Guidelines for managing these patients are needed. **Methods:** We conducted a detailed case series investigation of 4 HCV-positive patients with MM or a monoclonal gammopathy disorder. Patients were followed at the Mount Sinai Faculty Practice liver clinic. We also performed a detailed review of the literature exploring if there is any known association between HCV, MM, and monoclonal gammopathy. **Results and Conclusions:** There is no convincing evidence of a causal association between HCV and MM. HCV is linked to type II and type III cryoglobulinemia, specific kinds of monoclonal gammopathies of determinable significance. Whether a link exists between HCV and MM or monoclonal gammopathy of undetermined significance is unclear. Our case series provides the first evidence that modern HCV treatments with direct-acting antivirals can be safely and effectively co-administered with MM chemotherapy.

## Introduction

Approximately 2.7 million individuals in the United States are currently infected with the hepatitis C virus (HCV). Most were born between 1945 and 1965.^1^ As the baby boomer cohort ages, diseases typical of older patients are beginning to appear in HCV-positive patients, posing diagnostic and management challenges to hepatologists and other providers. These diseases include multiple myeloma (MM) and other disorders of plasma cells. Herein, we provide background information about MM, present several cases of HCV-positive patients with plasma cell disorders, and describe diagnostic strategies and management options.

MM is defined by the presence of more than 10% monoclonal plasma cells in the bone marrow (BM), as well as one or more of the following symptoms: hyper*c*alcemia, *r*enal insufficiency, *a*nemia, and/or *b*one lesions (“CRAB” symptoms). MM is thought to often be preceded by monoclonal gammopathy of undetermined significance (MGUS), an asymptomatic condition in which there is production of a small amount of monoclonal protein from a relatively small burden of monoclonal plasma cells.^2^ No known molecular or phenotypic markers distinguish MGUS and MM, and the genetic mutations in the abnormal antibody producing cells are largely the same.^3^ Characteristics of MM versus MGUS are delineated in Supplemental Table 1 (available at http://hic.sagepub.com/supplemental). Risk factors for MM include male sex, age over 55 years, African American race, obesity, and a family history of MM.^4^

## Methods

This is a case series review from December 2013 to June 2016. All cases were seen in the Mount Sinai Faculty Practice liver clinic. Cases are HCV-infected patients who also have a diagnosis of MGUS and/or MM. For historical comparison, we conducted a systematic literature search on PubMed to find all studies that examined HCV and MM/MGUS.

## Results and Discussion

### Pathophysiology

Abnormal responses to antigenic stimulation lead to chromosomal alterations, which in turn lead to the development of one or more premalignant clones derived from postgerminal center plasma cells, resulting in MGUS.^5,6^ Subsequent accumulation of additional genetic alterations leads to increased proliferation of the altered clone(s).^7^ The increased proliferation results in end-organ damage as the buildup of plasma cells reduces marrow function, and the excess immunoglobulins can cause complications such as myeloma cast nephropathy and hyperviscosity.^8-10^

HCV’s main effect is immune-mediated hepatocyte injury; however, chronic infection also increases the risk of hepatocellular carcinoma and certain lymphoproliferative disorders. HCV is most strongly associated with the non-Hodgkin’s B cell lymphomas^11,12^ and the mixed cryoglobulinemias, specifically type II and type III cryoglobulinemias (for a breakdown of the different cryoglobulinemias, see Supplemental Table 2; available at http://hic.sagepub.com/supplemental). HCV causes chronic antigenic B-cell stimulation and antibody production.^3^ While limited epidemiological studies have linked monoclonal plasma cell disorders with HCV, no direct pathophysiological proof links HCV and MM.^13-15^

### Epidemiology

MM is a relatively uncommon cancer, representing only 1% of all cancer diagnoses and 10% of all hematological malignancies. It is estimated that 0.7% of the US population will be diagnosed with MM, with 85% of diagnoses occurring in individuals 55 or older—an age range that a steadily increasing number of HCV patients in the United States and Europe are reaching.^4^ The well-known associations between HCV and other B-cell abnormalities support the possibility of an epidemiological link between HCV and MM; however, published data do not show a firm epidemiological association. A recent study conducted at Kaiser Permanente Southern California reported MM rates in patients with HCV versus those without HCV and found a relative risk (RR) of 3.41 (*P* < .0001). However, after adjusting for alcohol abuse, tobacco use, body mass index, and diabetes, the RR was no longer significant.^13^ An earlier meta-analysis of data from 5 studies did not find a significant association between HCV and MM.^14^ One case-control study in the meta-analysis reported an odds ratio of 4.2 in HCV-positive patients for developing MM.^15^ However, the data presented in this small study were not stratified for any established MM or cancer risk factors. Thus, the weight of the epidemiological evidence indicates that HCV infection is not a risk factor for MM. Nevertheless, even if there is no causal relationship between chronic HCV and MM, as the cohort of HCV-infected patients reaches the age of heightened MM risk, hepatologists and other clinicians are sure to see more patients affected by both diseases and will need guidance about how to manage them.

Below, we present 4 cases from the Mount Sinai Faculty Practice Liver Clinic. Each case involves HCV-positive patients of advancing age with either MGUS or MM. It is our hope that these cases offer guidance for hepatologists and other clinicians seeing similar patients who have concomitant diagnoses of HCV and MM/MGUS.

#### Case Presentation 1: Diagnostic work-up of monoclonal gammopathy in a human immunodeficiency virus and HCV co-infected patient

A 68-year-old Caucasian male with a history of well-controlled human immunodeficiency virus (HIV) on abacavir, lamivudine, and raltegravir (CD4 count 234, 19%, and an undetectable HIV viral load), chronic HCV, genotype 1a, HCV treatment naïve with cirrhosis (transient elastography score: 21 kPa) was diagnosed with MGUS in August 2014. The diagnosis was made as a result of tests carried out to investigate an elevated total protein-albumin (TPA) ratio. The patient had no complaints of CRAB symptoms, and a skeletal survey was negative for lytic lesions. The patient was referred to the myeloma clinic where a BM biopsy was discussed, but the patient declined. He was started on a 12-week course of sofosbuvir/ledipasvir (SOF/LDV) to treat his HCV in November 2014. He tolerated the antiviral course well and achieved a sustained virological response of 4 weeks (SVR4) in March before being lost to follow-up.

Asymptomatic monoclonal gammopathy encompasses both MGUS and smoldering multiple myeloma (SMM). SMM is distinguished from MGUS by a higher disease burden; however, both entities are clinically asymptomatic (Supplemental Table 1; available at http://hic.sagepub.com/supplemental). Patients should be referred to a hematologist for risk stratification of their disease, determination of follow-up intervals, and consideration for clinical trial participation. Follow-up should be life-long.^16^ Patients with MGUS have an estimated transformation rate to MM of 25% to 30% over 25 years.^2^ Several basic tests are used in the MGUS/MM work-up, including a serum protein electrophoresis, serum immunofixation, serum kappa/lambda chains, urine protein electrophoresis and urine immunofixation (Supplemental Tables 3 and 4; available at http://hic.sagepub.com/supplemental).

#### Case Presentation 2: Impact of HCV cure on a familiar type of monoclonal gammopathy for hepatologists, type II cryoglobulinemia

**Figure 1.**
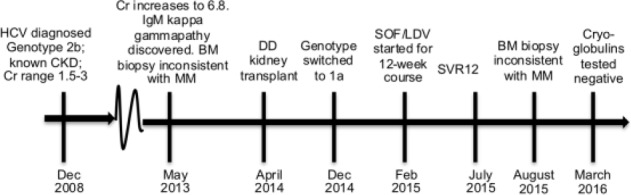
Timeline for Case 2. Abbreviations: HCV, hepatitis C virus; CKD, chronic kidney disease; Cr, creatinine; BM, bone marrow; MM, multiple myeloma; DD, deceased donor; SOF/LDV, sofosbuvir/ledipasvir; SVR12, sustained virological response at 12 weeks.

A 59-year-old diabetic male with HCV and biopsy-proven stage 1 fibrosis, a history of end-stage renal disease secondary to combined diabetic nephropathy, atherosclerotic disease, and cryoglobulinemic vasculitis, who had a deceased donor kidney transplant in April 2014 (see Figure 1), presented with symptomatic cryoglobulinemia while still on tacrolimus, mycophenolate mofetil, and prednisone (it was difficult to taper immunosuppressants due to problems of nonadherence and concerns centering on rejection). Symptoms of cryoglobulinemia included joint pain and a relapsing/remitting rash on his torso and arms. The patient had been diagnosed with genotype 2b HCV, received an HCV-positive kidney, switched to genotype 1a HCV after the transplant, and never received interferon treatment for HCV. The patient had an IgM kappa gammopathy (MGUS), first detected in May 2013 during an extensive work-up of a subacute profound worsening of renal function (creatinine increased from 2.5 to 6.8 in less than 9 months). While an IgM kappa gammopathy is not uncommon in cryoglobulinemia, especially type I and II cryoglobulinemia, given the patient’s worsening renal function at the time, it was necessary to rule out MM. BM biopsy performed shortly after the detection of the IgM kappa gammopathy showed a normocellular marrow with no plasma cell infiltrate. Kidney biopsy at the time showed advanced diabetic nephropathy with patchy interstitial fibrosis, tubular atrophy, and diffuse arteriosclerosis. Electron microscopy showed focal rare subepithelial deposits. In short, there was no pathological evidence of myeloma kidney at the time. The patient was followed in the myeloma clinic. The most recent BM biopsy in August 2015 showed plasma cells comprising only 3% to 5% of the overall cellularity with no excess of lambda or kappa chains. In mid-2015, the patient received a 12-week course of SOF/LDV and achieved an SVR12. In March 2016, cryoglobulins were retested and the patient was negative; however, the patient is still experiencing joint pain and neuropathy. This case illustrates the intersection of MGUS with cryoglobulinemia, a well-known extrahepatic manifestation of HCV. This patient received 12 weeks of SOF/LDV, resulting in an SVR12. He had resolution of his cryoglobulinemia and a negative M-spike after treatment, thereby resolving his monoclonal gammopathy of *determinable* significance.

#### Case Presentation 3: HCV treatment in a patient with active MM

A 60-year-old Caucasian male with chronic genotype 1a HCV infection, treatment naïve, with F3 fibrosis (transient elastography score: 12 kPa) presented with IgA-kappa MM with extensive lytic lesions in the skull, ribs, spine, sacrum, and left femur diagnosed in the winter of 2014-2015. There was no kidney involvement and no anemia at the time of MM diagnosis. The patient started a 12-week course of SOF/LDV 3 months after the monoclonal protein was initially discovered. Approximately 1 month after starting direct-acting antiviral (DAA) therapy, the patient was started on anti-myeloma chemotherapy with lenalidomide, bortezomib, and dexamethasone. He began monthly intravenous bisphosphonate due to the presence of the lytic lesions. Of note, the patient was on DAA therapy and chemotherapy at the same time for what amounted to a 7- to 8-week overlap (see Table 1). Overall, he tolerated his DAA and chemotherapy agents well, but he did develop mild bilateral upper extremity peripheral neuropathy, which was ascribed to be most likely from bortezomib. The patient completed 12 weeks of SOF/LDV and achieved SVR12. At last report, the patient had a complete response after 3 cycles of chemotherapy with no detectable M-spike in his serum or his urine.

**Table 1.** Direct-Acting Antiviral and Multiple Myeloma Chemotherapy Drug-Drug Interactions^a^.

	DEX	MEL	CYCLO	DOXO	THAL	LENA	POMA	BORT	CARF	PANO
LDV	C^b^	A	A	D^b^	A	A	A	A	C^b^	A
DAC	D^b^	A	A	D^b^	A	A	A	A	C^b^	A
EBV	A	A	A	A	A	A	A	A	A	A
VEL	C^b^	A	A	D^b^	A	A	A	A	C^b^	A
SOF	A	A	A	A	A	A	A	A	A	A
SMV	X^c^	A	A	D^c^	A	A	A	A	C^c^	C^c^
GZR	A	A	A	A	A	A	A	A	A	A
3-D	D^c^	A	A	D^c^	A	A	A	C^c^	A	D^c^
RBV	A	A	A	A	A	A	A	A	A	A

Abbreviations: DEX, dexamethasone; MEL, melphalan; CYCLO, cyclophosphamide; DOXO, doxorubicin; THAL, thalidomide; LENA, lenalidomide; POMA, pomalidomide; BORT, bortezomib; CARF, carfilzomib; PANO, panobinostat; LDV, ledipasvir; DAC; daclatasvir; EBV, elbasvir; VEL, velpatasvir; SOF, sofosbuvir; SMV, simeprevir; GZR, grazoprevir; 3-D, ombitasvir, dasabuvir, paritaprevir, and ritonavir; RBV, ribavirin.

a All drug-drug interactions run through Lexicomp drug interaction search engine.

A = No interaction.

B = The specified agents may interact, but there is little to no evidence of clinical concern.

C = Agents can be co-administered, but physicians should monitor therapy.

D = Consider therapy modification.

X = Co-administration not recommended.

b The direct-acting antiviral in question can increase serum levels of anti-myeloma agent.

c The chemotherapy agent in question can increase serum levels of the direct-acting antiviral.

While there are reports of cotreatment of MM and HCV during the interferon era, we are unaware of any literature looking at patients placed on both therapies in the DAA era. Table 1 reflects theoretical drug-drug interactions given what is known regarding metabolism of anti-HCV and MM drugs in the cytochrome p450 system.

As of the time of this writing, there are several classes of agents approved by the Food and Drug Administration to treat MM: proteasome inhibitors, traditional chemotherapeutic agents (eg, melphalan, cyclophosphamide, carmustine), histone deacetylase inhibitors (panobinostat), and immunotherapy (elotuzumab, daratumumab). MM remains an incurable malignancy. Patients are expected to relapse after their initial therapy, and subsequent therapy consists of additional lines of therapy utilizing agents from the classes mentioned above in combination. For a detailed description of relapsed and/or refractory MM, the reader is directed to excellent reviews of this topic.^17-19^ It is preferable to refer patients to centers specializing in the treatment of MM, as these centers have greater access to clinical trials incorporating novel therapies and therapeutic strategies.

Returning to the individual patient in case 3, the patient tolerated DAA treatment and chemotherapy well, with the exception of the development of peripheral neuropathy, most likely due to bortezomib.

#### Case Presentation 4: Stem cells harvested and stored for possible future use from a patient with MM and HCV, who was later cured of HCV

**Figure 2.**
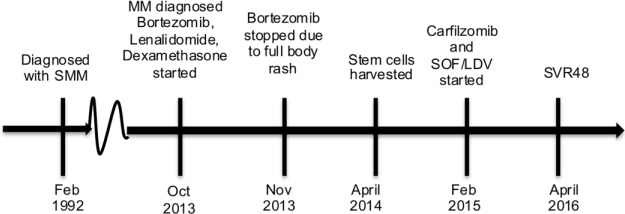
Timeline for Case 4. Abbreviations: SMM, smoldering multiple myeloma; MM, multiple myeloma; SOF/LDV, sofosbuvir/ledipasvir; SVR4, sustained virologic response for 4 weeks.

A 65-year-old Caucasian male with chronic genotype 1a HCV, stage F1 liver fibrosis (transient elastography score: 6.6 kPa), unsuccessfully treated with pegylated interferon and ribavirin over a decade previously, presented to liver clinic with a complex MM history (see Figure 2).

The patient was diagnosed in 1992 with SMM (BM biopsy was positive for 40% plasma cells, but he was asymptomatic without extra-marrow organ involvement at the time). In October 2013, the patient was found to have an elevation in his total protein to 10.7 and an increase in his M-spike to 4.72 (values had been 8.6 and 1.91, respectively, 1 year prior). The patient was complaining of rib pain at the time. A computed tomography scan of the chest showed lytic lesions and pathologic fractures of the ribs on the left side. Shortly afterward, the patient was started on bortezomib, lenalidomide, and dexamethasone. One month after starting chemotherapy, he developed a rash at the bortezomib injection site, which led to discontinuation of the agent. Patient was continued on lenalidomide and dexamethasone, which he tolerated well for several months. In March 2014, after 5 cycles of a lenaldomide-based regimen, the patient had an 85% reduction in his paraproteinemia, reflecting a successful response to therapy. Stem cells were harvested when the patient was still viremic with HCV in January 2015, his M-spike increased again, this time to 3.36. Carfilzomib was added to the patient’s chemotherapy regimen.

At this time, the patient was placed on 12 weeks of SOF/LDV concurrently with his carfilzomib, lenaldidomide, and dexamethasone. Patient achieved SVR48 as of April 2016. At last report, the patient had remission of his MM after several cycles of chemotherapy with no M-spike. Again, the case illustrates the fact that MM and HCV can be treated at the same time. Providers should of course be careful of drug-drug interactions (see Table 1) when combining treatments.

This case has an interesting complexity in that the patient had his stem cells harvested while he was still viremic with HCV, raising the concern that should his MM worsen again, he would need to undergo stem cell transplantation and his HCV may reoccur, prompting retreatment with DAAs. Recurrence of viremia after BM or solid organ transplantation is well described with many viruses, including, but not limited to, cytomegalovirus,^22^ Epstein-Barr virus,^23,24^ and hepatitis B.^25,26^ However, we were unable to find any reports of a patient auto-infecting oneself with HCV from previously harvested stem cells. If the patient were to undergo stem cell transplant in the future, the best course of action, in our expert opinion, would be to monitor HCV viral loads and to treat again with DAAs should viremia reoccur.

## Conclusion

As the HCV population ages, it will be important for hepatologists to be familiar with MGUS and MM. Primary care providers and hepatologists should know the basic warning signs and risk factors for MM, so that they can obtain appropriate initial data, and refer as necessary. Hepatologists should also know that while there is a paucity of data regarding the cotreatment of HCV and MM with modern therapies, simultaneous treatment is possible, as our case series illustrates, and appears to be safe for patients. Current evidence is inconclusive as to whether or not there is a link between HCV and MGUS/MM. More data are needed. However, it is our opinion that patients with HCV and MGUS/MM should be prioritized for DAA therapy.
